# Around the Hospitals

**Published:** 1893-07-29

**Authors:** 


### Around the Hospitals.

Notice to the Managers of Institutions.—Special space is now reserved for the insertion of notices of hospital meetings and festivals. The Editor requests that all notices may reach The Hospital Office, 428, Strand, at least one week before the date of each meeting.

**The London Fever Hospital.**—Fever hospitals, from their nature, are prevented from enjoying that amount of personal interest bestowed by the public on other medical charities. The report, however, of these hospitals usually affords some interest. In the record of the London Fever Hospital for 1892 it is interesting to note to what a low percentage deaths from scarlet fever can be brought. The mortality recorded is only 1·9 per cent. during the past year. Such figures are most reassuring, both in themselves and as compared with the percentage of deaths from this disease in London generally. The deaths from measles were more numerous, reaching 8·1 per cent., for although measles is regarded as a mild disease, statistics show that it is more fatal than many diseases of a severer nature. We think the two unfortunate sufferers from measles who contracted scarlet fever during their residence in the hospital were to be commiserated. Such cases form one of the awkward features of fever hospitals. where the various infectious cases are treated under the same roof. During the past year 748 patients were received; of these 593 were suffering from scarlet fever. During the year the drains at the hospital were found to be unsatisfactory, and the defects were promptly rectified with satisfactory results. The annual subscriptions amounted to £3,879, and donations to £3,227, both showing an increase on the previous year. We regard one clause in the privileges of the governors as being unfair to the institution. This clause provides that subscribers of one guinea who have subscribed for more than one year are entitled to send their servants for free treatment without respect to numbers. No employer should grudge a reasonable payment for treatment of a servant, and the public should not be encouraged to regard a small payment to an institution as adequately meeting the expenses of a long course of treatment such as scarlet fever, for instance, demands.

**The Devonshire Hospital and Buxton Bath Charity.**—This institution affords most invaluable aid to a class of patients to whom help can seldom be rendered by other hospitals, and its situation adapts it especially to the rheumatic and pulmonary affections treated there. During the past year 2,612 in-patients were received. Of this number 2,306 cases were of rheumatic or gouty character, and all these can be said to have received decided benefit from the treatment received. Comparatively few cases of phthisis apply for admission, although the rules of the charity are especially framed to admit sufferers from this disease when not in the last stages. The dry air of Buxton renders the hospital so suited to this complaint that it is a pity more advantage is not taken of the benefits afforded by the institution, which, besides having natural advantages, possesses an admirable staff of workers zealous to promote the comfort of the patients and the interests of the institution. At the last annual meeting the Rev. R. Reed remarked that not a single complaint had been made by any of the patients during the past year. We are glad to learn that the number of annual subscribers has increased during the same period.

